# Measuring Unintentional Force Drift: Reliability, Number of Trials, and Age-Related Differences

**DOI:** 10.3390/s26185912

**Published:** 2026-09-18

**Authors:** Michał Pawłowski

**Affiliations:** Institute of Sport Sciences, Department of Human Motor Behavior, Academy of Physical Education in Katowice, 40-065 Katowice, Poland; m.pawlowski@awf.katowice.pl

**Keywords:** visual feedback, isometric force control, measurement error, intraclass correlation coefficient

## Abstract

Unintentional force drift (UFD) describes the decline in force that occurs when visual feedback is removed despite the intention to maintain a target force. This study determined the reliability of UFD, the number of trials required for reliable assessment, and whether reliability differed across age groups. Twenty-four healthy adults completed six trials of simultaneous bilateral upper extremity force production task (isometric elbows flexion at 20% of Maximal Voluntary Contraction under visual and no visual feedback). Relative and absolute reliability of force and force drift were assessed using intraclass correlation coefficient (ICC), standard error of measurement and minimal detectable changes. Overall, measurement reliability improved as the number of trials increased. Force showed good reliability with visual feedback and excellent reliability without visual feedback. Force drift showed moderate reliability with visual feedback and good reliability without visual feedback (ICC = 0.84). Across all participants, five trials provided a UFD measurement with good reliability. UFD measurement properties differed across age groups, with older adults showing lower ICC values, greater measurement error, and requiring more trials than younger adults. In conclusion, five trials are recommended for UFD assessment, although more trials may be required in older adults.

## 1. Introduction

Maintaining stable force control during object manipulation is essential for many everyday activities, such as carrying a box or a plate of food while looking around. The central nervous system (CNS) must therefore regulate force primarily using proprioceptive and tactile feedback throughout task performance. Effective regulation is essential for the safe and precise performance of everyday tasks, whereas impaired force control may reduce the ability to perform these tasks independently, particularly with advancing age.

Accurate force regulation without continuous visual feedback can be examined experimentally using isometric force production tasks. In these tasks, participants first match their produced force to a target using continuous real-time visual feedback, which enables for immediately detection and correction of performance errors [1]. Once force has stabilized, visual feedback is removed while the instruction to maintain the target force remains unchanged. Under these conditions, force output typically begins to decline despite the continued intention to maintain it [2,3,4]. This unintended reduction in force is defined as unintentional force drift (UFD), and its magnitude can be quantified as the difference in force between the beginning and end of the period without visual feedback [5,6,7]. UFD has subsequently been documented in single-finger tasks [8], one- and two-hand finger-force production [9], isometric knee extension [10], foot force-matching task [11] and bilateral hand force-matching tasks [4]. However, these consistent findings at the group level do not show whether UFD can be measured reliably in individual participants. Furthermore, assessing UFD requires consistent measurements across repeated trials. Measurement reliability describes the consistency of repeated measurements obtained under comparable conditions [12,13,14]. It comprises two distinct but complementary components: relative and absolute reliability [12,15]. Relative reliability indicates whether differences between participants remain consistent across repeated measurements, whereas absolute reliability describes the magnitude of measurement error within individual participants. These components provide complementary information because high relative reliability can coexist with substantial absolute measurement error [14,15].

Despite the growing use of UFD in experimental research, it remains unknown whether its magnitude can be measured reliably in the individual participants. Furthermore, the number of trials required for a reliable UFD measurement is still unclear. Therefore, the aim of the present study is to determine the measurement reliability of UFD during a bilateral isometric force production task.

Previous studies have shown that force oscillates close to the prescribed target when visual feedback is available, whereas its absence consistently leads to UFD across single- and multi-effector tasks, including unilateral and bilateral force production [9,16,17,18]. Studies using steady-state force production tasks have shown that force-related outcomes can be reproduced across trials or sessions. However, their reliability depends on the outcome measured and the number of included repetitions [19,20,21]. These observations suggest that both maintained force and UFD may provide reproducible information about differences between participants. Therefore, the first research question was whether force and force drift were consistent across consecutive trials and could be measured reliably with and without visual feedback. It was hypothesized that both outcomes would remain stable across trials and show at least good level of reliability across all conditions.

The number of trials used to quantify UFD has varied widely across studies. Previous protocols included two or three repetitions per task condition [3,5,16], four trials without visual feedback at each target level [2], four or six repetitions depending on the protocol [9], six trials per condition or target level [4,6,10], and 12-trial blocks or 13 repetitions per condition [8,18]. These differences make studies difficult to compare and leave it unclear whether UFD measurements based on different numbers of repetitions are equally reliable. Accordingly, the second research question was how many trials were required for reliable UFD measurement. Based on the protocols described above, it was hypothesized that at least six trials would be required to achieve satisfactory reliability.

Reliable UFD measurement is also important for age-related comparisons because force control changes across the lifespan. Older adults generally exhibit greater force fluctuations and reduced steadiness during submaximal isometric contractions, although the magnitude of these age-related differences depends on the muscle group, force level, task constraints, and availability of visual feedback [22,23,24]. Because measurement reliability is affected by within-participant variability, these differences may reduce the reproducibility of UFD measurements. However, it is unclear whether UFD reliability differs across age groups. The third research question was whether the reliability of force and force drift measurements differed among young, middle-aged, and older adults. It was hypothesized that the reliability of both outcomes would decline with advancing age.

## 2. Materials and Methods

### 2.1. Participants

Twenty-four healthy adults (13 women and 11 men) participated in this study. Participants were recruited through a public invitation, with a mean age of 36.0 ± 19.0 years, a body height of 173.1 ± 10.4 cm, and a body mass of 70.5 ± 9.3 kg. For age-related analyses, participants were classified into three groups: young adults (n = 10, 18.8 ± 0.4 years), middle-aged adults (n = 8, 35.9 ± 6.9 years), and older adults (n = 6, 64.8 ± 3.3 years). All participants were right-handed according to self-report. Eligibility was restricted to individuals who reported engaging in fewer than 6 h of planned physical activity per week and who did not regularly participate in organized amateur or competitive sport. Planned physical activity was defined as any intentionally scheduled form of exercise or physical training. The usual weekly frequency and duration of physical activity were assessed by self-report during the eligibility interview; no standardized questionnaire or activity monitoring device was used. This criterion was applied to limit the potential influence of sport-specific skills and adaptations on bilateral force production. Additional eligibility criteria included the absence of current musculoskeletal injuries affecting the upper extremities and neurological disorders. No participants were excluded from the analysis. The study was conducted in accordance with the principles of the Declaration of Helsinki and was approved by the Institutional Review Board (approval No. 4-VII/2024). Written informed consent was obtained from all participants before the experimental procedures.

### 2.2. Equipment

Force was measured using a custom-built dynamometer equipped with two uniaxial load cells (model 060-P665-01, Honeywell Sensotec, Columbus, OH, USA), with one load cell independently recording the force generated by each upper extremity. Signals from both load cells were acquired synchronously at 1500 Hz using a DTS Desktop data acquisition system and the manufacturer’s software (MyoResearch, Noraxon Inc., Scottsdale, AZ, USA). The forces recorded from both extremities were summed to obtain the total bilateral force. During visual feedback, the total force was displayed in real-time on a computer monitor.

### 2.3. Experimental Procedure

**Figure 1:**
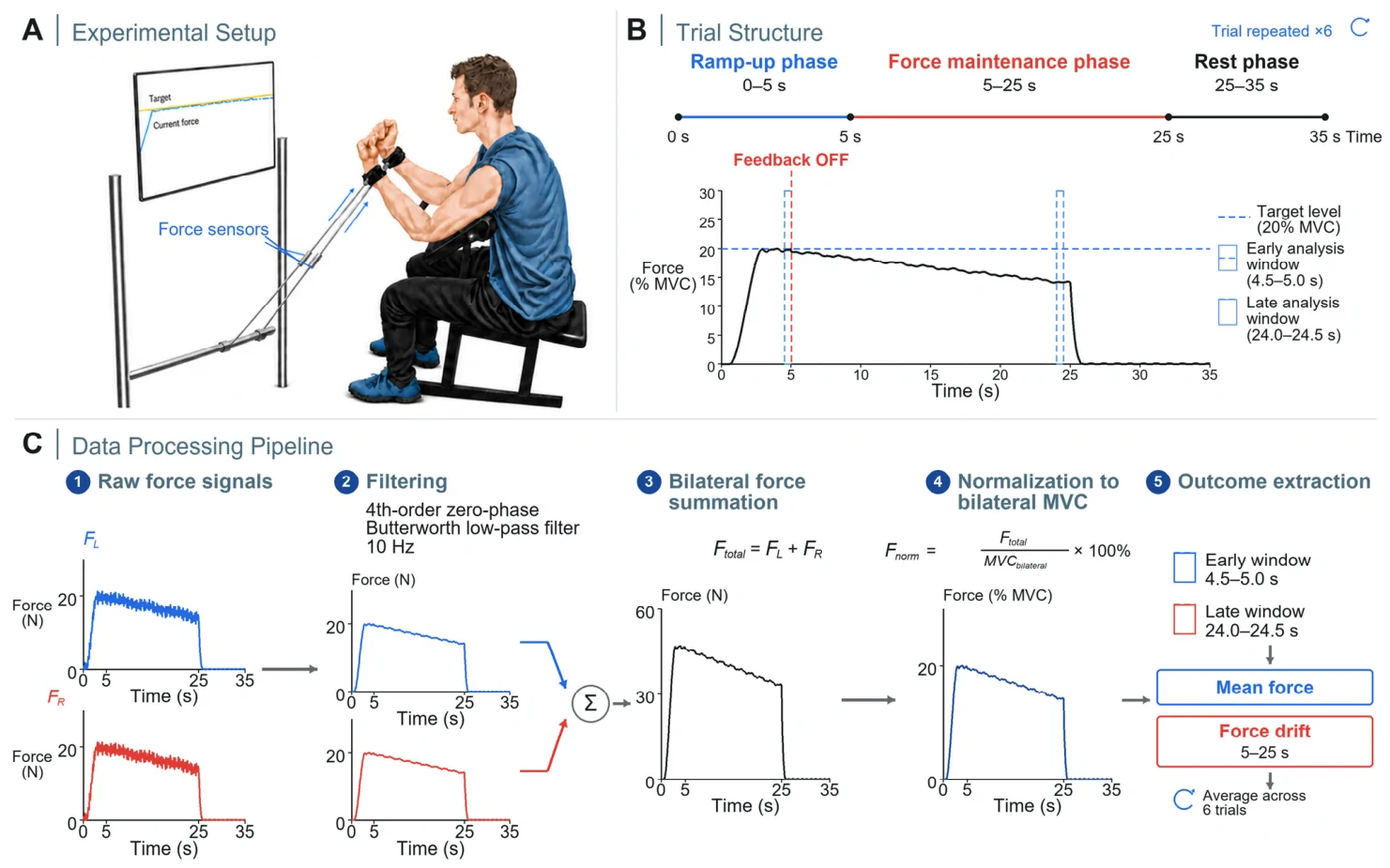
Schematic overview of the experimental workflow for assessing the reliability of bilateral force production and UFD. (**A**) Experimental setup showing the custom dynamometer used to measure isometric elbow flexion forces with and without visual feedback. (**B**) Structure of a single experimental trial consisting of a ramp-up phase (0–5 s), a force maintenance phase (5–25 s), and a rest phase (25–35 s). In the no-feedback condition, visual feedback was removed after the first 5 s of force production. The target force corresponded to 20% of Maximal Voluntary Contraction (MVC). (**C**) Data processing pipeline. Raw force signals from both load cells were low-pass filtered (fourth-order Butterworth, 10 Hz), summed to bilateral force, normalized to bilateral MVC, and used to calculate the outcome variables: mean force and force drift. Force drift was determined from the change in force between the early (4.5–5.0 s) and late (24.0–24.5 s) analysis windows. Outcome measures were averaged across six trials for each experimental condition.

Participants were seated in a custom-built dynamometer according to the experimental setup illustrated in Figure 1A. The chest was supported against the Scott bench pad, while both upper extremities rested on the arm support. The seat height, spacing between the dynamometer arms and the distance from the bench to the dynamometer were adjusted for each participant to ensure comfort and consistent elbow position. Dynamometer handles were attached around both forearms immediately proximal to the wrist joints, allowing for simultaneous bilateral measurement of isometric elbow flexion force. A computer monitor positioned 1 m in front of the participant at eye level displayed the target force and the total bilateral force generated in real-time (when visual feedback was available).

Before data collection, participants completed a practice to become familiar to the dynamometer and visual feedback. Maximal Voluntary Contraction (MVC) was subsequently performed: three maximal bilateral isometric elbow flexions. Each repetition lasted 3 s and was followed by 30 s of rest. Standardized verbal encouragement was provided during each maximal contraction. The highest total bilateral force recorded across the three repetitions was defined as MVC and used to calculate the target force in the task, corresponding to 20% MVC. A tolerance range of ±1% MVC was defined around the target force, such that force values between 19% and 21% MVC were considered to represent a successful target.

The experimental task consisted of six consecutive isometric bilateral elbow flexions in each of two experimental conditions (with and without visual feedback). Participants were instructed to reach the target force as quickly as possible within the first 5 s and then maintain it throughout the whole force production period. Each trial consisted of three consecutive phases: ramp-up from 0 to 5 s, force maintenance from 5 to 25 s, and rest from 25 to 35 s. Auditory signals indicated the beginning and end of the 25 s force production period (see Figure 1B). Both conditions were performed in the randomized order. In the visual feedback condition, real-time feedback of total bilateral force remained available throughout the trial. In the without feedback condition, feedback was removed 5 s after trial started. In all trials, participants reached the predefined target range of 19–21% MVC within the initial 5 s period; therefore, no trial was repeated or excluded because of inaccurate initial force matching. Participants were then instructed to maintain the same force level for the remaining 20 s without visual feedback. Feedback was restored during the rest phase once the total bilateral force returned to zero. A minimum rest period of 3 min was provided between conditions.

### 2.4. Data Processing

Force signals recorded during the MVC assessment and experimental trials were processed offline using MATLAB (R2025b, MathWorks, Inc., Natick, MA, USA). Signals from each load cell were low-pass filtered using a fourth-order, zero-phase Butterworth filter with a cut-off frequency of 10 Hz and summed to obtain total bilateral force. The highest total bilateral force recorded across the three MVC trials was defined as bilateral MVC. Force recorded during the experimental trials was normalized to this value and expressed as a percentage of MVC (%MVC). Mean force and force drift were calculated separately for each trial and experimental condition. Mean force was defined as the average normalized bilateral force during the 20 s force-maintenance phase (5–25 s). Force drift was calculated using two 0.5 s analysis windows. The early force value (t_1_) was the mean force recorded between 4.5 and 5.0 s, immediately before removing visual feedback. The late force value (t_2_) was the mean force recorded between 24.0 and 24.5 s. Force drift was calculated as:

(1) $$Force\;Drift=\frac{F\left(t_{2}\right)-F\left(t_{1}\right)}{F\left(t_{1}\right)}\;\times \;100$$

### 2.5. Statistical Analysis

Statistical analyses were performed using Statistica 13 (TIBCO Inc., Palo Alto, CA, USA). Statistical significance was set at *p* < 0.05. The assumptions of the parametric analyses were checked. When the assumption of sphericity was violated, the Greenhouse–Geisser correction was applied. Bonferroni-adjusted comparisons were performed where appropriate, and effect sizes for analyses of variance were reported as partial eta squared (η_p_^2^). One-way repeated-measures analyses of variance (rmANOVAs) were conducted within each experimental condition to determine whether either outcome changed systematically across consecutive trials.

Measurement reliability was evaluated using complementary relative and absolute indices, as reproducibility and measurement error represent distinct properties of repeated measurements [14,25]. Relative reliability was assessed using the intraclass correlation coefficient (ICC). Selection of the ICC form followed established distinctions among the statistical model, consistency versus absolute agreement, and single versus average measurements [26,27,28,29]. Relative reliability was assessed separately for mean force and force drift in each experimental condition using a two-way mixed-effects, consistency, average-measure ICC_(3,k)_:

(2) $${ICC}_{3,k}=\frac{MS_{B}-MS_{E}}{MS_{B}}$$

where MS_B_ and MS_E_ are the mean squares of the rmANOVA and k denotes the number of repeated trials. Ninety-five percent confidence intervals (95% CI) were calculated for all ICC estimates. The magnitude of ICC values was interpreted according to the recommendations of Koo and Li [29]: poor (<0.50), moderate (0.50–0.75), good (0.75–0.90) and excellent (>0.90). Absolute reliability was quantified using the standard error of measurement (SEM), calculated as:

(3) $$SEM=SD\sqrt{1-ICC}$$

the minimal detectable change at the 95% confidence level (MDC_95_) [14], calculated as:

(4) $$M{DC}_{95}=1.96*SEM*\sqrt{2}$$

and trial-to-trial variability (TTV). TTV was calculated separately for each participant as the mean absolute difference between consecutive trials (Trials 2–1, 3–2, 4–3, 5–4, and 6–5). Group TTV was subsequently calculated as the mean of the individual participant values. TTV was expressed in the same units as the corresponding outcome: % MVC for mean force and percentage points for force drift. Because measurement reliability may depend on the number of trials included [20,30], reliability was evaluated using averages of one to six trials. The expected reliability for an increasing number of trials was estimated separately for each outcome and experimental condition using the Spearman–Brown formula [12]. The minimum number of averaged trials required to achieve ICC values of 0.50, 0.75, and 0.90 was subsequently determined. All reliability calculations were performed separately for each outcome and experimental condition and were subsequently repeated within each age group.

## 3. Results

**Figure 2:**
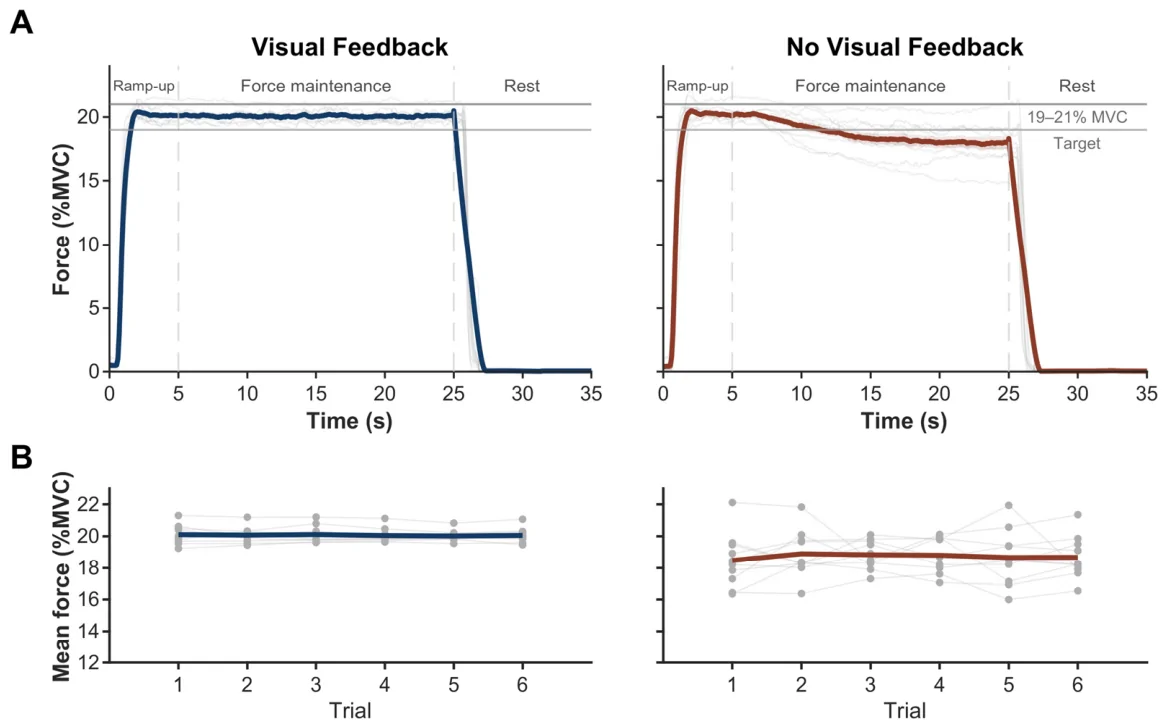
General characteristics of the isometric force maintenance task performed with and without visual feedback. (**A**) Representative force profiles showing the group mean, standard error and individual force traces observed during trials performed with visual feedback (**left**) and without visual feedback (**right**). (**B**) Mean force across six consecutive trials in the visual feedback (**left**) and no-visual-feedback (**right**) conditions. Thick lines indicate group means and thin gray lines represent individual participants.

As an overall characterization of task performance, isometric force production was examined under conditions with and without visual feedback (Figure 2). Representative force traces show that participants reached the target force level in each condition. However, UFD during the force maintenance phase was observed only when no visual information was provided on the screen (Figure 2A). Despite this expected qualitative difference, rmANOVA revealed no significant effect of trial number on force in either the feedback (F_(5, 115)_ = 1.03; *p* = 0.402, η_p_^2^ = 0.043) or no-feedback condition (F_(5, 115)_ = 0.522; *p* = 0.759, η_p_^2^ = 0.022) (Figure 2B). No significant changes were observed across consecutive trials.

### 3.1. Reliability of UFD

**Figure 3:**
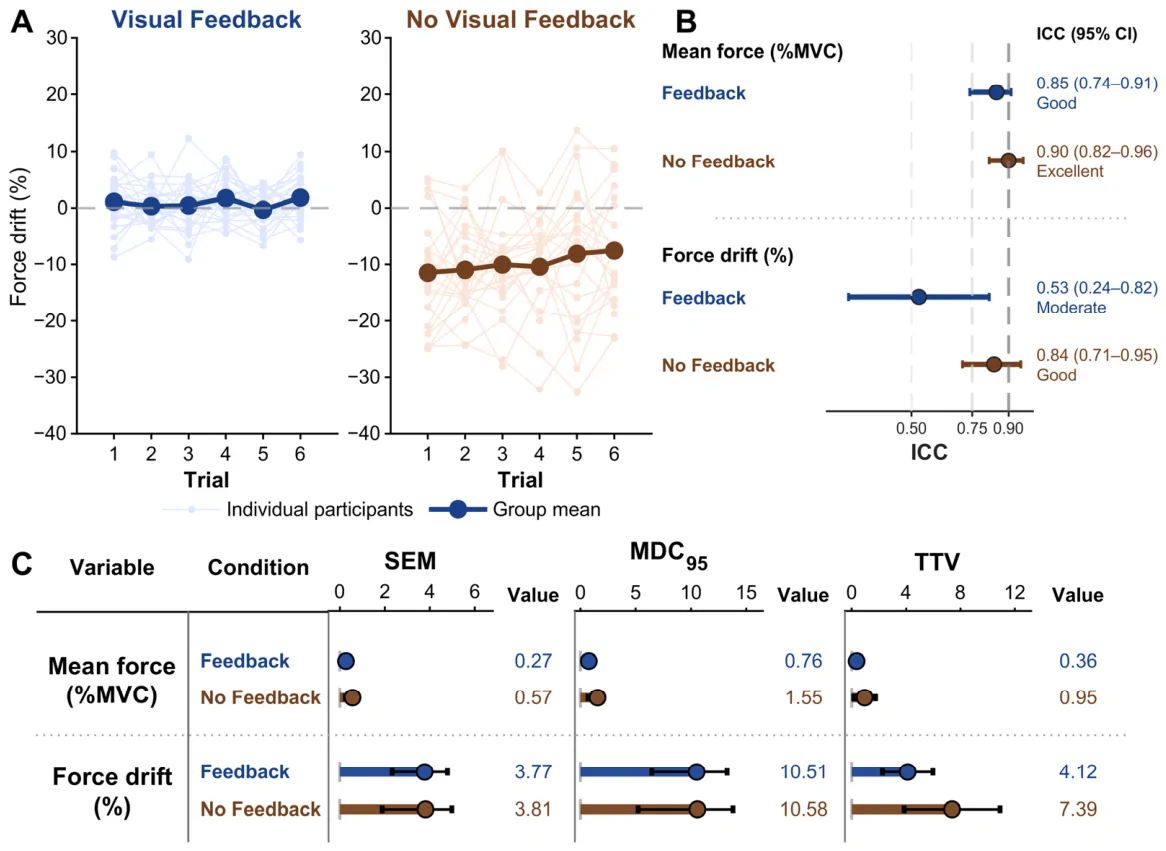
Reliability of force and UFD across repeated trials. (**A**) Force drift across six consecutive trials in the visual-feedback (**left**) and no-visual-feedback (**right**) conditions. Thin lines represent individual participants, and thick colored lines represent group means. (**B**) Relative reliability of mean force and force drift, quantified using intraclass correlation coefficients (ICCs) with 95% confidence intervals. Points indicate ICC values, and horizontal bars indicate the corresponding confidence intervals. (**C**) Absolute reliability of mean force and force drift, quantified using the standard error of measurement (SEM), minimal detectable change at the 95% confidence level (MDC_95_), and trial-to-trial variability (TTV) in both experimental conditions. For SEM and MDC_95_, horizontal bars represent intervals derived from the ICC confidence limits; for TTV, they represent ±1 SD. Mean-force outcomes are expressed as percentages of Maximal Voluntary Contraction (%MVC), whereas force drift outcomes are expressed as percentages relative to force at the beginning of the force-maintenance phase.

As shown in Figure 2B, mean force remained stable across the six consecutive trials in both the visual feedback and no-visual-feedback conditions, with no significant effect of trial number. Force drift was similarly stable across repeated trials, with rmANOVA showing no significant effect of trial number in either the visual feedback condition (F_(5, 115)_ = 1.544; *p* = 0.182, η_p_^2^ = 0.063) or the no-visual-feedback condition (F_(5, 115)_ = 1.208; *p* = 0.31, η_p_^2^ = 0.05). Although individual participants demonstrated TTV, these fluctuations showed no consistent direction and did not produce systematic changes in force drift across repeated measurements (Figure 3A). Mean force showed good reliability in the visual-feedback condition (ICC = 0.85, 95% CI: 0.74–0.91) and excellent reliability in the no-visual-feedback condition (ICC = 0.90, 95% CI: 0.82–0.96). Force drift presented moderate reliability with visual feedback (ICC = 0.53, 95% CI: 0.24–0.82) and good reliability without visual feedback (ICC = 0.84, 95% CI: 0.71–0.95) (Figure 3B). In aspect of absolute reliability, mean force, SEM, MDC_95_, and TTV were 0.27, 0.76, and 0.36% MVC in the visual-feedback condition and 0.57, 1.55, and 0.95% MVC in the no-visual-feedback condition, respectively. For force drift, the corresponding values were 3.77, 10.51, and 4.12% with visual feedback and 3.81, 10.58, and 7.39% without visual feedback, respectively (Figure 3C).

### 3.2. Minimum Number of Trials Required for Reliable UFD Assessment

**Figure 4:**
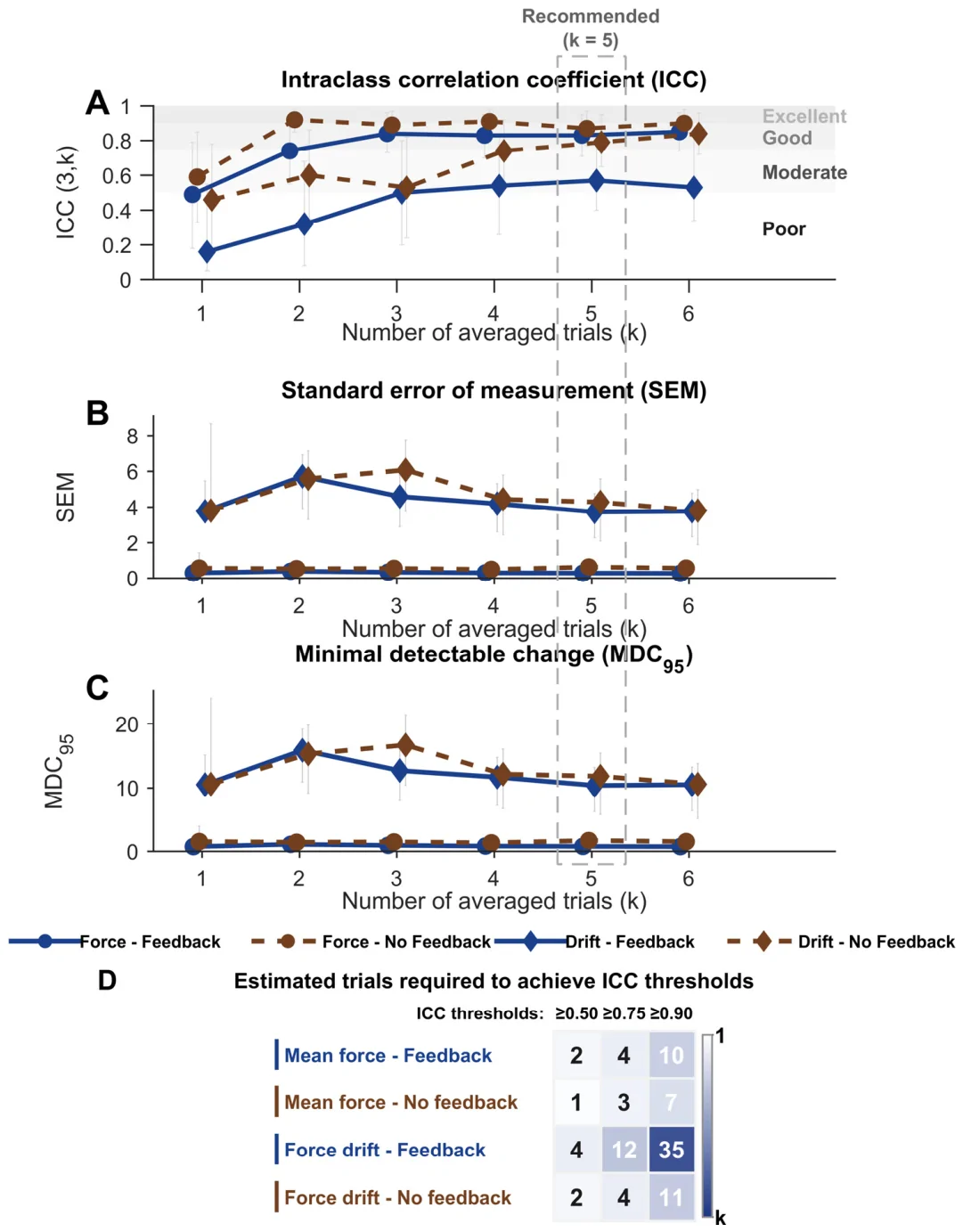
Influence of number of trials on the reliability of UFD measurements. (**A**) Intraclass correlation coefficients (ICC_(3,k)_) for mean force and force drift as a function of the number of trials included in the analysis under visual feedback and no visual feedback. Shaded regions indicate conventional ICC reliability levels. (**B**,**C**) Corresponding changes in the standard error of measurement (SEM) and minimal detectable change at the 95% confidence level (MDC_95_). For SEM and MDC_95_, mean-force values are expressed as % MVC and force drift values as percentage points. (**D**) Estimated number of trials required to achieve predefined ICC thresholds of 0.50, 0.75, and 0.90 for each outcome and experimental condition.

The number of trials required for reliable UFD assessment was examined by recalculating relative and absolute reliability for results based on one to six trials (Figure 4). For UFD condition without feedback, a single trial showed poor relative reliability, whereas analyses based on two and three trials remained within the moderate-reliability range. With four trials, the ICC reached the boundary between moderate and good reliability, consistent with the minimum predicted by the Spearman–Brown formula. When five and six trials were included, ICC values remained clearly within the good-reliability range and showed little further change, indicating greater stability of the reliability estimate (Figure 4A).

After reaching their highest values for the two trials, SEM and MDC_95_ generally decreased as additional trials were included, with only minor changes between five and six trials (Figure 4B,C). The estimated number of trials required to reach predefined ICC thresholds differed between outcomes and experimental conditions (Figure 4D). For UFD, the Spearman–Brown formula indicated that 2, 4, and 11 trials were required to achieve ICC values of 0.50, 0.75, and 0.90, respectively. For mean force, the corresponding estimates were 2, 4, and 10 trials in the visual-feedback condition and 1, 3, and 7 trials in the no-feedback condition. Force drift measured with visual feedback showed the lowest relative reliability and was estimated to require 4, 12, and 35 trials to reach the respective thresholds. Thus, four trials represented the estimated minimum for reaching the good-reliability threshold for UFD, whereas estimates based on five and six trials showed greater stability.

### 3.3. Age-Related Differences in UFD Reliability

**Figure 5:**
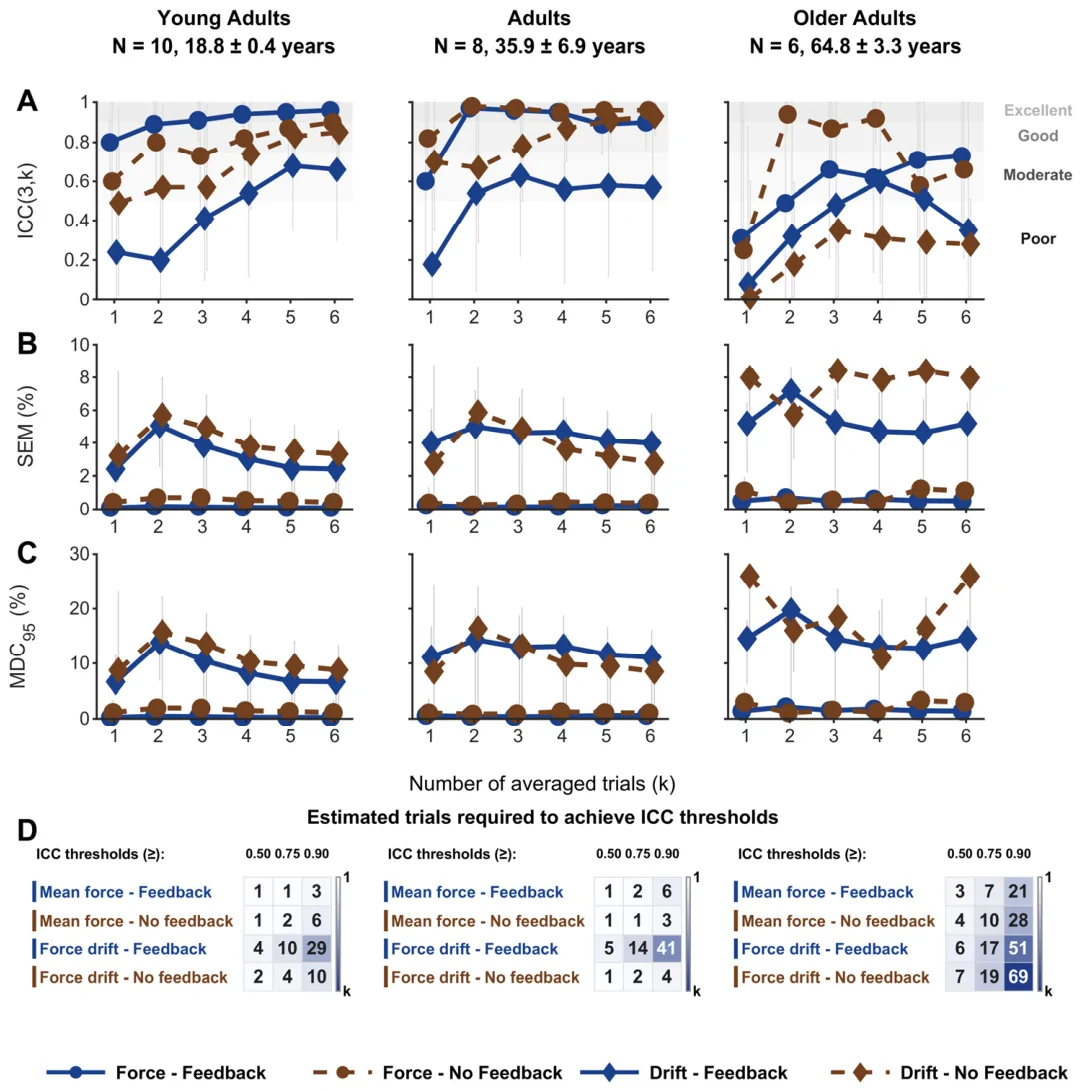
Age-related differences in the reliability of force and force drift measurements. Columns present results for young adults, adults, and older adults, respectively. (**A**) Intraclass correlation coefficients (ICC_(3,k)_) for mean force and force drift as a function of the number of trials under and without visual feedback. Shaded regions marked ICC reliability levels. (**B**,**C**) Corresponding standard errors of measurement (SEM) and minimal detectable changes at the 95% confidence level (MDC_95_). Error bars represent 95% confidence intervals. For SEM and MDC_95_, mean-force values are expressed as % MVC and force drift values as percentage points. (**D**) Estimated numbers of trials required to achieve predefined ICC thresholds of 0.50, 0.75, and 0.90 for each outcome, experimental condition, and age group. Darker shading indicates a larger trial requirement.

Reliability was subsequently examined separately in young, middle-aged, and older adults to determine whether the reproducibility of force-production outcomes differed across age groups (Figure 5). Age-specific reliability profiles differed more for force drift than for mean force. In young and middle-aged adults, greater number of trials generally increased ICC values and reduced SEM and MDC_95_ for both outcomes. In older adults, mean-force reliability also improved with consecutive trials, although more trials were generally required to reach the predefined ICC thresholds. The largest differences were observed for UFD in the no-visual-feedback condition. The reported numbers of trials required to achieve ICC values of 0.50, 0.75, and 0.90 were 2, 4, and 10 in young adults; 1, 2, and 4 in middle-aged adults; and 7, 19, and 69 in older adults, respectively (Figure 5D). Consistent with these results, older adults showed lower and less stable ICC values together with higher SEM and MDC_95_ for UFD. Thus, the age-related differences in reliability were comparatively modest for mean force but substantially greater for UFD, particularly in older adults.

## 4. Discussion

The present study provides a comprehensive assessment of the within-session reliability of UFD that considers both the number of repeated trials and participant age. Mean force remained stable across consecutive trials and showed high reliability under both visual and no visual feedback. UFD measured following the removal of visual feedback also demonstrated good within-session relative reliability, although its larger measurement error indicated substantial within-participant variability. Reliability improved as more trials were included. An estimate based on four trials reached the threshold for good relative reliability, while results based on five or more trials remained consistently within the good level. The measurement properties of UFD also differed across age groups, with older adults showing lower relative reliability, greater measurement error, and requiring more trials to reach comparable reliability thresholds.

The reproducibility of UFD raises the question of which processes underlie the decline in force without visual feedback. Early studies attributed this phenomenon to the limited capacity of visuomotor memory, proposing that the internal representation of target force progressively loses accuracy once visual information is removed [2]. This interpretation received indirect support from observations of greater force drop in participants with Parkinson’s disease and the activity of prefrontal regions during memory-guided force control [31,32]. However, later studies showed that force drift was not caused simply by time spent without visual feedback. Force decreased when participants continuously produced force, but not after an equivalent period of rest or during intermittent force production [6,16]. Working-memory limitations may therefore influence task performance, particularly during cyclic force production, but they do not provide a sufficient explanation for UFD during constant force production. An alternative explanation is provided by the referent configuration hypothesis (RC), according to which isometric force emerges from the difference between the actual and referent configurations of the acting effector [33,34,35]. When movement toward the referent configuration is mechanically constrained, this difference results in force production. The RC hypothesis proposes that an unintentional drift of the referent configuration toward the actual configuration progressively reduces this difference and consequently lowers the generated force. Relevant sensory information allows the effects of this drift to be corrected, whereas removal of visual feedback makes the drift visible as a gradual reduction in force. This behavior has been interpreted as a natural relaxation process within the physiological system involved in force production [5,6,16].

The dynamic nature of UFD has direct consequences for its measurement. UFD is a difference-based outcome calculated from force recorded within two brief time windows at different stages of a trial; variability in either estimate therefore contributes to its final magnitude. Averaging force within each window reduces the influence of temporal fluctuations but cannot eliminate differences between trials in initial force stabilization or in the onset, time course, and magnitude of the subsequent drift. Variation in UFD may therefore reflect both measurement error and short-term differences in how the same participant regulates force across repeated trials. Comparable differences have been reported in studies of isometric force production, in which variables derived from the same force-signal showed different levels of reproducibility depending on the outcome and temporal phase examined [19,36]. Other studies have further demonstrated that the stability of force depends on the number of observations included in their calculation [20,21], while repeated practice may itself alter force steadiness before performance stabilizes [37]. Measurement reliability should therefore be interpreted separately for each outcome and in relation to how that outcome is calculated and how many trials are included, rather than being generalized to the force-production task as a whole. In the present study, the ICC obtained for UFD without visual feedback indicated that differences between participants accounted for a substantial proportion of its total variability. This did not imply identical values across repeated measurements: an estimate based on a single trial showed poor relative reliability, which improved only when additional trials were added.

The observed dependence of UFD reliability on the number of performed trials helps resolve an important methodological inconsistency in the UFD literature. Previous studies have quantified UFD using different numbers of repetitions, ranging from two or three trials [3,5,16] and four trials [2] to six [4,6,9,10] or even 12–13 trials [8,18]. Trial number has therefore largely been selected on the basis of individual experimental protocols rather than empirical evidence concerning measurement reliability. For the present bilateral force production task, five trials represent a pragmatic choice between measurement reliability and procedural efficiency. Five trials are therefore recommended for the present protocol, although the number required may differ with the task, force level, outcome calculation, and population examined [20,21].

The relevance of the population examined is underscored by the age-related differences in UFD reliability observed in the present study. Classic studies have shown that older adults exhibit greater force fluctuations during submaximal isometric contractions and these changes are associated with age-related alterations in motor unit behavior [22,38,39]. Subsequent evidence has demonstrated that the magnitude of these differences depends on the muscle group, force level, task demands, and availability of visual feedback [23,24,40]. In the present study, mean force remained highly reliable across age groups, whereas UFD showed lower ICC values, greater SEM and MDC_95_, and higher trial requirements with advancing age. Lower ICC values should not be interpreted in isolation as evidence of poorer force control, because ICC reflects the magnitude of between participant differences relative to within-participant measurement error. However, their convergence with greater absolute measurement error indicates that individual UFD estimates were less reproducible among older participants. Age-specific trial estimates derived from the Spearman–Brown formula should be interpreted with caution. In older adults, the estimated requirements of 7, 19, and 69 trials to reach ICC values of 0.50, 0.75, and 0.90, respectively, extend beyond the six trials performed and therefore represent mathematical extrapolations. These estimates do not provide a sufficiently robust basis for specifying trial requirements or practical recommendations. Instead, they indicate that the corresponding reliability thresholds were not reached within the examined range of trials and provide a descriptive indication of comparatively lower within-session repeatability in the older subgroup. Studies involving larger samples and more repeated trials are needed before recommendations for this group can be established. This presents a practical challenge because increasing the number of trials may improve measurement precision while simultaneously increasing assessment duration and testing demands. The consequences of prolonged testing are likely to depend on both individual capacity and the physical and cognitive demands of the task [41]. Experimental mental fatigue has not been shown to impair force steadiness consistently during simple upper extremity isometric tasks [42,43]. However, force fluctuations can increase when force production is performed concurrently with a demanding cognitive task, with this effect being particularly evident in older adults [44]. This distinction suggests that attentional demands imposed during the measurement itself may be more relevant than mental fatigue induced before testing. Protocols intended for older and clinical populations should therefore minimize testing demands without compromising measurement precision. Potential modifications, including shorter force production periods, longer recovery intervals, or adaptive trial number criteria require independent validation because they may alter the magnitude or temporal development of UFD.

Several limitations should be considered in the present study. First, reliability was assessed across repeated trials within a single experimental session The present findings therefore characterize within-session reliability and do not establish the stability of UFD across separate days or testing sessions. UFD may be influenced by factors such as fatigue, motivation, and attention, which may vary between measurement sessions. Second, the age groups were relatively small, particularly the older group, and subgroup specific ICC outcomes and trial requirements should therefore be interpreted carefully. Third, the findings are specific to healthy adults performing bilateral isometric elbow flexion at 20% MVC and may not generalize to other effectors, force levels, task durations, or clinical populations. Furthermore, because the forces produced by both upper extremities were summed, the present analysis could not determine whether participants redistributed force between the dominant and non-dominant extremities while maintaining a similar total force output. Such redistribution may represent an important aspect of bilateral force control following the removal of visual feedback. Although symmetry indices can describe the relative contribution of each extremity, a more comprehensive assessment requires analysis of how the two forces covary to stabilize the task-level performance. Future studies could therefore examine interlimb coordination using approaches such as the uncontrolled manifold framework [45,46], which can distinguish between variability that preserves total bilateral force and variability that changes it. Finally, physical fatigability, perceived effort, and attentional state were not measured, preventing their potential contributions to trial-to-trial variability from being determined.

In conclusion, the present findings demonstrate that UFD magnitude can be estimated with good within-session relative reliability during bilateral isometric force production, provided that the estimate is based on multiple trials. A single trial was insufficient to characterize individual UFD reliably, whereas five averaged trials provided a stable and practically achievable estimate under the present protocol. Measurement properties nevertheless differed across age groups, indicating that a procedure suitable for the general adult sample may not provide equivalent precision in older adults. The proposed five-trial protocol should therefore be regarded as a practical reference for bilateral force production at 20% MVC. Further studies involving larger samples, clinical populations, and alternative task durations are required to determine whether the protocol can be simplified while retaining adequate measurement precision.

## Data Availability

The data supporting the findings of this study are available from the corresponding author upon reasonable request.

## Abbreviations

The following abbreviations are used in this manuscript:

CI	Confidence Interval
CNS	Central Nervous System
ICC	Intraclass Correlation Coefficient
MDC	Minimal Detectable Change
MVC	Maximal Voluntary Contraction
rmANOVA	Repeated Measures Analysis of Variance
SD	Standard Deviation
SEM	Standard Error of Measurement
TTV	Trial-To-Trial Variability
UFD	Unintentional Force Drift
